# Correction to “Mitogen‐Activated Protein Kinase Inhibition Augments the T Cell Response Against *HOXB7*‐Expressing Tumor Through Human Leukocyte Antigen Upregulation”

**DOI:** 10.1111/cas.70094

**Published:** 2025-05-06

Komatsuda H, Wakisaka R, Kono M, et al. Mitogen‐activated protein kinase inhibition augments the T cell response against *HOXB7*‐expressing tumor through human leukocyte antigen upregulation. *Cancer Sci*. 2023;114:399–409. doi: 10.1111/cas.15619

In the above article, there were errors in the following texts:

From Section 3.2:

Based on computer‐based algorithms, *HOXB7*_8‐25_ (PLLLKLLKSVGAQKD) was selected as a potential candidate for eliciting antigen‐specific HTL responses.

The correct text should be as follows:

Based on computer‐based algorithms, *HOXB7*_8‐25_ (NTLFSKYPASSSVFATGA) was selected as a potential candidate for eliciting antigen‐specific HTL responses.

From Section 4:

As *HOXB7*_18‐26_ (SSVFAPGAF) might bind to HLA‐A*26:01 in in silico analysis, the elongation of *HOXB7*_8‐25_ to *HOXB7*_8‐26_ would improve the antigenicity of the peptide by inducing both HTLs and CTLs.

The correct text should be as follows:

As *HOXB7*_18‐26_ (SSVFATGAF) might bind to HLA‐A*26:01 in in silico analysis, the elongation of *HOXB7*_8‐25_ to *HOXB7*_8‐26_ would improve the antigenicity of the peptide by inducing both HTLs and CTLs.

We apologize for these errors.

